# Cytoplasmic MSH2 Related to Genomic Deletions in the *MSH2/EPCAM* Genes in Colorectal Cancer Patients With Suspected Lynch Syndrome

**DOI:** 10.3389/fonc.2021.627460

**Published:** 2021-05-14

**Authors:** Lin Dong, Shuangmei Zou, Xianglan Jin, Haizhen Lu, Ye Zhang, Lei Guo, Jianqiang Cai, Jianming Ying

**Affiliations:** ^1^ Department of Pathology, National Cancer Center/National Clinical Research Center for Cancer/Cancer Hospital, Chinese Academy of Medical Sciences and Peking Union Medical College, Beijing, China; ^2^ Department of Pathology, Peking University Shenzhen Hospital, Shenzhen, China; ^3^ Beijing Microread Genetics, Beijing, China; ^4^ Department of Hepatobiliary Surgery, National Cancer Center/National Clinical Research Center for Cancer/Cancer Hospital, Chinese Academy of Medical Sciences and Peking Union Medical College, Beijing, China

**Keywords:** DNA mismatch repair, Lynch syndrome, colorectal cancer, genetic testing, immunotherapy

## Abstract

**Background:**

A large proportion of patients with Lynch syndrome (LS) have MSH2 abnormalities, but genotype-phenotype studies of *MSH2* mutations in LS are still lacking. The aim of this study was to comprehensively analyze the clinicopathological characteristics and molecular basis of colorectal cancer (CRC) in patients with uncommon MSH2 cytoplasmic expression.

**Methods:**

We retrospectively reviewed 4195 consecutive cases of CRC patients diagnosed between January 2015 and December 2017 at the Cancer Hospital Chinese Academy of Medical Sciences. Of the 4195 patients with CRC, 69 were indicated to have abnormal MSH2 expression through tumor immunohistochemical staining. Genetic tests, such as next-generation sequencing, large genomic rearrangement (LGR) analysis, microsatellite instability status analysis and genomic breakpoint analysis, were performed. Clinicopathological and molecular characteristics and clinical immunotherapy response were analyzed.

**Results:**

Forty-five of 69 patients were identified to have LS with pathogenic germline mutations in *MSH2* and/or *EPCAM*. Of these LS patients, 26.7% were confirmed to harbor large genomic rearrangements (LGRs). Of note, three tumors from two unrelated family pedigrees exhibited a rare cytoplasmic MSH2 staining pattern that was found in LS patients with *EPCAM/MSH2* deletions. RNA analysis showed that two novel mRNA fusions of *EPCAM* and *MSH2* resulted in the predicted protein fusion with MSH2 cytoplasmic localization. Analyses of genomic breakpoints indicated that two novel deletions of *EPCAM* and *MSH2* originated from Alu repeat-mediated recombination events. Our study also provides clinical evidence for the beneficial effect of the PD-1 inhibitor pembrolizumab for CRC patients that exhibit cytoplasmic MSH2 staining.

**Conclusion:**

Our study demonstrates that the rare cytoplasmic MSH2 staining pattern should be fully recognized by pathologists and geneticists. Given the specific genotype-phenotype correlation in LS screening, we advocate that all CRC patients with cytoplasmic MSH2 staining in histology should be screened for LGRs of *EPCAM* and *MSH2*.

## Introduction

Lynch syndrome (LS), an autosomal dominant hereditary disorder, is the most common colorectal cancer (CRC) predisposition syndrome, accounting for 1%–3% of all newly diagnosed CRCs (1). LS is caused by pathogenic germline mutations in one of several dMMR genes (*MLH1*, *MSH2*, *MSH6* and *PMS2*) and deletions in *EPCAM* (2–4). Deficient DNA mismatch repair (dMMR) is defined as a lack of immunohistochemically detectable MMR protein expression in tumors and microsatellite instability (MSI), and it is the diagnostic hallmark of LS (5). Concurrent loss of MSH2 and MSH6 proteins, which can be revealed by a universal reflex testing program using immunohistochemistry (IHC), is a common dMMR expression pattern that generally indicates the presence of a germline *MSH2* mutation (6, 7). In addition, deletions of the 3’ end of *EPCAM* are thought to lead to tissue-specific epigenetic silencing of *MSH2* through aberrant promoter methylation (2). *EPCAM* deletions account for approximately 20% of cases in which MSH2 and/or MSH6 are lost but there is no detectable *MSH2* germline mutation (8–10). These unique cases cannot be distinguished from those in which *MSH2* mutations are revealed by IHC analysis of MMR proteins (11). Multiplex ligation-dependent probe amplification (MLPA) analysis, which is used to detect large genomic rearrangements (LGRs), is a complementary diagnostic tool in comprehensive genetic testing strategies for LS (12, 13).

IHC analysis of MMR proteins is a cost-effective initial screening method for LS (14, 15). A previous study suggested that the protein expression pattern of MSH2 and MSH6 proteins can be categorized into three types: intact staining of both proteins, loss of both proteins, and isolated loss of MSH6 (16). Mutations in *MSH2* are generally thought to result in the loss of IHC-detectable MSH2 and MSH6. Some challenging cases present with loss of MSH2 and with patchy loss of MSH6, as reported by Dr. Pearlman (17). Cytoplasmic staining is commonly interpreted as having no known significance, with previous literature citing questionable IHC staining quality (14). Dr. Sekine delineated a cryptic nonfunctional in-frame EPCAM-MSH2 fusion protein resulting from a genomic rearrangement between *EPCAM* intron 5 and *MSH2* intron 2 in one LS patient with aberrant cytoplasmic MSH2 localization in colon cancer (18).

Mismatch repair status has been widely used as a positive predictive marker for clinical benefit of immune checkpoint blockade approved by US Food and Drug Administration in metastatic CRCs with dMMR or MSI-high (19). Immunotherapy treatment becomes a new and promising therapeutic option for advanced CRC patients. The importance of accurate interpretation of MMR protein IHC has been paid more attention by clinicians and pathologist (20, 21).

Unfortunately, no other studies are available that might shed light on whether this observation is simply an artifact or a valid finding in some *MSH2*-related LS cases. Due to this uncertainty, it is necessary to systematically assess rare cytoplasmic MSH2 abnormalities to avoid missing potential LS probands and to stratify CRC patients for immunotherapy. In this study, we investigated clinicopathological characteristics and performed molecular characterizations of MSH2 abnormalities in a large cohort of 4195 CRC patients with a particular focus on elucidating the association of cytoplasmic MSH2 staining with genotype in real-world LS patients.

## Materials And Methods

### Selection of Cases

Among 4195 eligible patients from the **C**olo**r**ectal Cancer **I**nitiative **S**creening **P**rogram for **L**ynch **S**yndrome (**CRISPLS**) in the Cancer Hospital of the Chinese Academy of Medical Sciences between January 2015 and December 2017, we identified a cohort of 69 patients with loss of the MSH2 and/or MSH6 proteins who had been screened by IHC staining for tumor MMR proteins. Detailed information on the CRISPLS cohort was previously reported (22). Clinicopathological characteristics and information about cancer personal/family history were collected for patients from the CRISPLS cohort who had undergone surgical resection and for whom a sufficient DNA sample was available. The study was approved by the Ethics Committee of NCC/CICAMS (NCC1790). Individual informed consent was waived because of the retrospective nature of the study. Patients were informed if they were identified as having LS.

### Immunohistochemistry Analysis

IHC analyses of MMR proteins, including MLH1, PMS2, MSH2, MSH6 and BRAF V600E, were routinely performed in CRC patients. One representative block of formalin-fixed, paraffin-embedded tumor tissue was selected per patient. Monoclonal antibodies against MLH1 (clone ES05), PMS2 (clone EPR3947), MSH2 (clone FE11), MSH6 (clone EP49) (Beijing Zhongshan Golden Bridge Biotechnology, China), and BRAF V600E (VE1) (Ventana Medical Systems, AZ, USA) were used. Briefly, after deparaffinization, rehydration and antigen-retrieval, 4-μm-thick sections were stained in a Ventana Benchmark IHC automated slide stainer and visualized using the OptiView DAB IHC detection kit (Ventana Medical Systems). The absence of nuclear staining in tumor cells or very faint nuclear staining in focal tumor cells was defined as loss of protein expression (abnormal staining). Stromal/lymphoid cells and nearby normal glandular epithelium of the bowel served as positive internal controls.

### PCR-Based Microsatellite Instability Analysis

Microsatellite instability (MSI) testing was performed on tumor and normal DNA using a fluorescence PCR-based assay (MSI-Reader MSI Analysis System; MICROREAD, Beijing, China) in which six mononucleotide repeat markers (NR-21, NR-24, NR-27, BAT-25, BAT-26 and MONO-27) and two pentanucleotide repeat loci (Penta-C and Penta-D) were amplified to confirm the identity of paired tumor and benign tissues. The PCR products were run on an Applied Biosystems 3500 Genetic Analyzer and analyzed using GeneMapper v5.0 software (Applied Biosystems, CA, USA). Tumors with shifts in two or more markers were classified as unstable MSI-high (23, 24).

### Isolation of Genomic DNA

Formalin-fixed, paraffin-embedded tumors and adjacent normal tissue were collected from the cohort. Genomic DNA was extracted using a TGuide Genomic DNA One-Step Kit and a TGuide Automated Nucleic Acid Preparation Instrument (TIANGEN BIOTECH, Beijing, China) according to the manufacturer’s instructions as previously described (22).

### Germline Mutation Testing by Targeted Next-Generation Sequencing

Next-generation sequencing technology was performed with the Agilent SureSelect-XT Low Input Target Enrichment kit (Agilent Technologies, CA, USA) for germline mutation testing of *MMR* genes from genomic DNA extracted from normal FFPE samples according to the manufacturer’s instructions. Molecular barcoded DNA libraries were hybridized with a commercial ClearSeq Inherited Disease multigene panel that covered total exons and intron boundaries within at least ±20 bases of the *EPCAM*, *MLH1*, *PMS2*, *MSH2* and *MSH6* genes (Agilent Technologies). A detailed protocol for variant annotation and classification was described previously (22, 25). Diagnosis of LS is dependent on identifying the pathogenic germline variants of MMR genes. Interpretations of germline variants are classified according to the database of the International Society of Gastrointestinal Hereditary Tumors (InSiGHT) and guideline of the American College of Medical Genetics and Genomics (ACMG). The carriers with likely pathogenic or pathogenic variants are defined as LS patients.

### Multiplex Ligation-Dependent Probe Amplification (MLPA)

Large genomic rearrangements (LGRs) in *MSH2* and *EPCAM* genes among MSH2-deficient patients with no germline mutations identified by next-generation sequencing were assessed by MLPA using the SALSA MLPA P003 *MLH1/MSH2* kit (including the 3’ end of *EPCAM*) and P072 *MSH6* kit (including the *EPCAM/MSH2* region) (MRC-Holland, Amsterdam, The Netherlands). Fragment analysis of amplified genomic DNA extracted from normal FFPE samples was performed on an ABI3500 capillary sequencer (Applied Biosystems). The MLPA data were quantitatively analyzed using Coffalyser.Net software (www.mlpa.com).

### Reverse Transcription PCR and Amplification of *EPCAM-MSH2* Fusion Transcripts

Total RNA was extracted from the peripheral blood leukocytes of patients using TRIzol reagent (Agilent Technologies). cDNA was synthesized using a PrimeScript II 1^st^ Strand cDNA Synthesis Kit (Takara, Japan) and analyzed for *EPCAM*-*MSH2* fusion transcripts. Polymerase chain reaction (PCR) products were loaded directly on 2% agarose gels and visualized under UV illumination. Selected PCR products were sequenced on an ABI 3500xl capillary DNA analyzer (Applied Biosystems, CA, USA). Details of the PCR primers used are provided in **Table S1**.

### Analysis of Breakpoint Mapping

A series of long-range PCR experiments designed to span the putative deletion region were performed to characterize the exact breakpoints in the *EPCAM* and *MSH2* genes using a TaKaRa LA PCR Kit (Takara, Japan) according to the manufacturer’s protocol. The PCR products were analyzed by electrophoresis on ethidium bromide-stained 1% agarose gels and then subjected to UV detection. The expected fragment was purified and sequenced on an ABI 3500xl capillary DNA analyzer (Applied Biosystems). Details of the PCR primers are provided in **Table S2**.

### Statistical Analysis

A univariate analysis of categorical variables was performed by cross tabulation using a chi-square test to compute p-values. An unpaired t test was used for continuous variables. Statistical descriptions or analyses were conducted using SPSS (Version 22; SPSS Inc., Chicago, IL, USA) or Prism (Version 7; San Diego, CA, USA) software. All tests were 2-tailed, and p-values < 0.05 were considered statistically significant.

## Results

### Clinicopathological Characteristics of the Study Cohort

We retrospectively enrolled a consecutive cohort of 4195 CRC patients. Among these patients, 345 were eligible, exhibiting dMMR, and 69 exhibited abnormal MSH2 protein expression (**Figure S1**). The frequency of MSH2 deficiency (dMSH2) among the dMMR group was 20% (69 of 345). The demographic and clinical characteristics of patients with MSH2-deficient CRC are summarized in **Table 1**. Briefly, the mean age was 50 years at diagnosis of CRC (standard deviation, 12.7), 94.2% was aged 70 years or younger, 65.2% was male, 47.8% occurred in the proximal colon, 46.4% was at tumor stage II, adenocarcinoma was most common histological type (91.3%). The differences in age of onset, personal history of cancer and family history of LS-related cancers between LS and dMSH2 were statistically significant.

**Table 1:** Clinicopathological characteristics of patients with MSH2-deficient CRC.

		LS		Inconclusive dMSH2		*x^2^*/*t*	*P*-value
		n = 45		n = 24			
		No.	Percent	No.	Percent		
Age at diagnosis (years)							
Mean (SD)			48.3 (11.3)		53.3 (14.9)	–	0.120
≤70		45	100.0%	20	83.3%	-	0.012
>70			0.0%	4	16.7%		
Gender						3.757	0.053
Male		33	73.3%	12	50.0%		
Female		12	26.7%	12	50.0%		
Tumor location						3.202	0.362
Proximal colon		20	44.4%	13	54.2%		
Distal colon		9	20.0%	4	16.7%		
Rectum		11	24.4%	7	29.2%		
Other^a^		5	11.1%		0.0%		
Tumor stage						3.326	0.505
I		9	20.0%	4	16.7%		
II		20	44.4%	12	50.0%		
III		8	17.8%	6	25.0%		
IV		5	11.1%		0.0%		
NA		3	6.7%	2	8.3%		
Histological type						0.594	0.743
Adenocarcinoma		41	91.1%	22	91.7%		
Mucinous adenocarcinoma		3	6.7%	2	8.3%		
Other^b^		1	2.2%		0.0%		
Personal history of cancers						5.269	0.022
Yes		15	33.3%	2	8.3%		
No		30	66.7%	22	91.7%		
Family history of LSRC						11.829	0.003
Yes		30	66.7%	6	25.0%		
No		15	33.3%	17	70.8%		
Unknown			0.0%	1	4.2%		
Revised Bethesda guidelines						2.406	0.121
Met		40	88.9%	17	70.8%		
Not met		5	11.1%	7	29.2%		
Not available							
Amsterdam II criteria						7.134	0.028
Met		9	20.0%		0.0%		
Not met		36	80.0%	23	95.8%		
Not available			0.0%	1	4.2%		

dMSH2, deficient MSH2; LSRC, Lynch syndrome-related cancers; SD, standard deviation.

a. Ungrouped data, including 5 patients with 4 CRC tumors located in two sites among the proximal colon, distal colon or rectum, and 1 colon tumor with an unspecified location.

b. Indicates two synchronous cancers at the sigmoid colon and splenic flexure with mucinous carcinoma and adenocarcinoma histologic types, respectively.

### Clinicopathological Characteristics of LS Patients With Genomic Rearrangement of *MSH2/EPCAM*

To estimate the frequency and specificity of *MSH2* germline mutations among patients with CRC in the real world, we systematically analyzed a consecutive CRC patient cohort that had been universally screened for LS in our previous study (22). Germline analyses were performed on samples from 69 patients with MSH2-deficient CRC. The frequencies of *MSH2/EPCAM* mutations among different categories of CRC subgroups are presented in detail in **Table S3**. Forty-five patients (1.1%) were identified as having LS with pathogenic germline mutations in *MSH2* and/or *EPCAM*. Of these, 12 (26.7%) were confirmed to carry LGRs in *MSH2/EPCAM* by MLPA, including six probands with *MSH2* genomic deletions, four with *MSH2-EPCAM* deletions (two cases from a family pedigree), and two with *EPCAM* deletions. The clinicopathological and molecular findings for these 12 patients with LGRs are presented in **Table 2**. All cases were identified as microsatellite instability-high (MSI-H) by PCR-MSI. Notably, all 12 of these patients were also ascertained to have a strong cancer family history. Patients with *MSH2/EPCAM* LGRs exhibited an earlier age of CRC onset (mean: 43.8 years) than those with LS with *MSH2* SNV/indel (mean: 49.9 years).

**Table 2:** Clinical, pathological and molecular findings in CRC tumors with *MSH2* LGR-associated LS.

CaseNo.	Age, y/Sex	MMR loss	MSI status	MSH2 abnormal location	Tumor location	Stage	Gene of LGR	NGS result	MLPA result	mRNA result	LS	Family history
49	61/M	MSH2- MSH6-	MSI-H	No	Proximal colon and rectum	II	*MSH2*	Neg	*MSH2* -16 del	ND	Yes	Yes
90	44/M	MSH2- MSH6-	MSI-H	No	Distal colon	I	*MSH2*	Neg	*MSH2* Exon 7 del	ND	Yes	Yes
100	54/M	MSH2- MSH6-	MSI-H	No	Proximal colon	IV	*MSH2 EPCAM4*		*EPCAM* Exon 9 and *MSH2* Exon 1-14 del	ND	Yes	Yes
164^#^	17/F	MSH2- MSH6-	MSI-H	Cytoplasmic staining	Rectum	IV	*MSH2 EPCAM*	Neg	*EPCAM* Exon 3-9 and *MSH2* Exon 1 del	*EPCAM-MSH2* fusion	Yes	Yes
165	53/M	MSH2- MSH6-	MSI-H	No	Proximal colon	III	*EPCAM*	Neg	EPCAM Exon 9 del	ND	Yes	Yes
168	37/M	MSH2- MSH6-	MSI-H	No	Distal colon	II	*MSH2 EPCAM*	Neg	*EPCAM* Exon 9 and *MSH2* Exon 1-14 del	ND	Yes	Yes
205	48/F	MSH2- MSH6-	MSI-H	No	Rectum	II	*MSH2*	Neg	*MSH2* Exon 3-8 del	ND	Yes	Yes
212	60/M	MSH2- MSH6-	MSI-H	No	Rectum	I	*MSH2*	Neg	*MSH2* Exon 8-11 del	ND	Yes	Yes
234	28/M	MSH2- MSH6-	MSI-H	No	Rectum	I	*MSH2*	Neg	*MSH2* Exon 3 del	ND	Yes	Yes
271^#^	45/M	MSH2- MSH6-	MSI-H	Cytoplasmic staining	Distal colon	IV	*MSH2 EPCAM*	Neg	*EPCAM* Exon 3-9 and *MSH2* Exon 1 del	*EPCAM-MSH2* fusion	Yes	Yes
318	49/M	MSH2- MSH6-	MSI-H	No	Rectum	I	*MSH2*	Neg	MSH2 Exon 1-3 del	ND	Yes	Yes
345	27/F	MSH2- MSH6-	MSI-H	Cytoplasmic staining	Proximal colon	III	*EPCAM*	Neg	*EPCAM* Exon 3-9 del	*EPCAM-MSH2* fusion	Yes	Yes

Del, deletion; F, female; IHC, immunohistochemistry; LGR, large genomic rearrangement; LS, Lynch syndrome; M, male; MLPA, multiplex ligation-dependent probe amplification; MMR, mismatch repair; MSI, microsatellite instability; Neg, negative; NGS, next-generation sequencing; No., number; ND, not done. # indicates that the patient is from a family pedigree shown in **Figure 2A**.

### Aberrant Cytoplasmic Localization of the MSH2 Protein Among LS Patients

**Figure 1:**
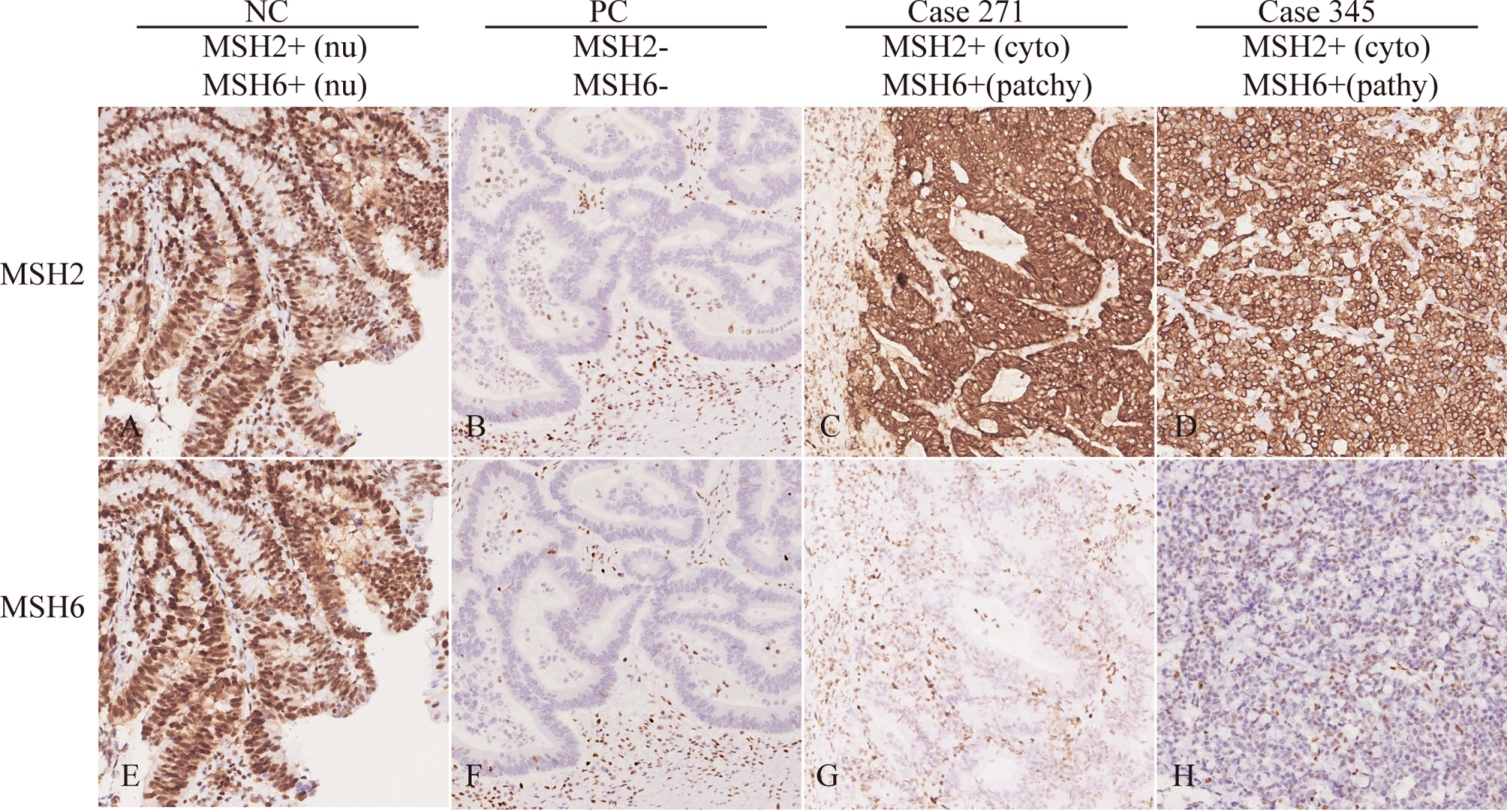
Rare MSH2 cytoplasmic staining in CRC. IHC of MSH2 and MSH6 in selected patients showing protein expression in tumor cells. The NC (negative control with proficient MMR) showed nuclear staining of MSH2 and MSH6. The PC (positive control with deficient MMR) showed loss of nuclear staining of MSH2 and MSH6. However, patient 271 and 345 showed cytoplasmic MSH2 and patchy/weak MSH6 nuclear staining in tumor cells. Nu, nuclear; cyto, cytoplasmic.

**Figure 2:**
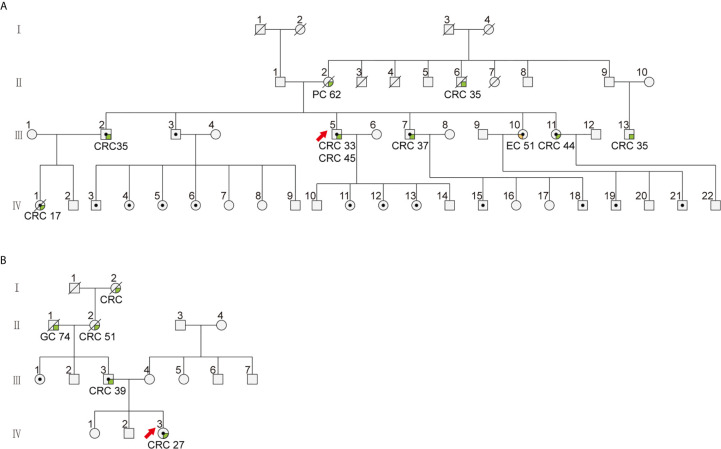
The family pedigree of patients with cytoplasmic staining of MSH2 in colorectal cancer. The family pedigrees of **(A)** Patient 271 (III5) and 164 (IV1) and **(B)** patient 345 (IV3) investigated in this study. The proband is indicated by the red arrow. The black dots indicate mutation carriers confirmed by genetic testing. The cancer types and age of onset are listed beneath each affected family member.

MSH2 abnormalities usually manifest as the absence of nuclear staining in tumor cells. Among the 69 patients with an MSH2 abnormality, we noted that three (4.3%) exhibited rare cytoplasmic MSH2 localization in tumor cells but showed patchy expression of the MSH6 protein that was somewhat weaker in tumor cells than in internal control cells (patient 164 and patient 271 from one family pedigree and patient 345) (**Figures 1** and **S2**). They presented a classical family history of LS cancer (**Figure 2**). PCR-MSI tests indicated a status of MSI-high in the tumors of these patients (**Figure S2**). MLPA tests identified combined deletions of *MSH2* and *EPCAM* in all three patients. Two patients had a heterozygous large genomic deletion in *EPCAM* (exons 3–9) and *MSH2* (exon 1). One patient harbored heterozygous deletion of exons 3–9 of *EPCAM* (**Figure S4**).

### Alu-Mediated *EPCAM/MSH2* Cytoplasmic MSH2 Related to Genomic Deletions in the MSH2/EPCAM Genes in Colorectal Cancer Patients with Suspected Lynch Syndrome Rearrangement Is Responsible for Cytoplasmic Localization of MSH2 in LS Patients

**Figure 3:**
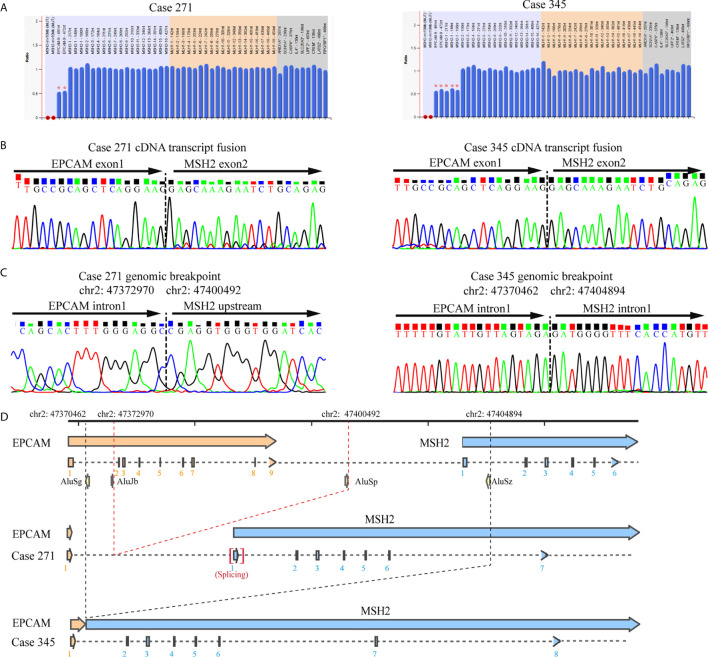
Identification of the germline *EPCAM-MSH2* fusion in patients with suspected LS and cytoplasmic MSH2 staining. **(A)** MLPA testing identified the presence of a large genomic deletion in the *MSH2* and *EPCAM* genes in patients 271 and 345. **(B)** Sanger sequencing of *EPCAM* and *MSH2* transcripts amplified by the E1M23-1F and E1M23-2R primer pairs in the peripheral blood cDNA of the proband revealed a fusion between exon 1 of *EPCAM* and exon 2 of *MSH2*. **(C)** Sequencing analysis of long-range PCR products from genomic DNA defined the exact breakpoint, as indicated by the genomic locus. **(D)** Schematic representation showing the genomic deletions mediated by recombination between Alu elements.

To elucidate the molecular characteristics of cytoplasmic localization of MSH2 in tumors, we performed mapping analysis of gene fusions and gene breakpoints for two individual patients. Sanger sequencing of cDNA from the blood lymphocytes of the patients suggested that a fusion transcript of EPCAM and MSH2 was the source of aberrant MSH2 localization in tumors. Sequencing revealed a common fusion of exon 1 of EPCAM and exon 2 of MSH2 in patient 271 and patient 345. On the basis of this fusion transcript analysis, we performed a series of long-range PCR experiments on the region of interest between intron 1 of EPCAM and intron 1 of MSH2 to identify the precise breakpoint in the *MSH2/EPCAM* LGRs (**Figures S6** and **S7**). Ultimately, we mapped the breakpoint to intron 1 of EPCAM (hg38, chr2:47372970)/MSH2 upstream [hg38, chr2:47400492]) in patient 271 and to intron 1 of EPCAM (hg38, chr2:47370462)/intron 1 of MSH2 [hg38, chr2:47404894]) in patient 345. Both were novel mutations that were not reported in the International Society for Gastrointestinal Hereditary Tumours (InSiGHT) variant database (http://insight-database.org). Repeated Alu elements are implicated in the etiology of genomic rearrangement for many inherited cancers (26). Our analysis using RepeatMasker (http://www.repeatmasker.org) revealed that these breakpoints lay within Alu elements that share high sequence identities. A schematic diagram is shown in **Figure 3**.

### Response to Treatment With a PD-1 Inhibitor in a Colon Cancer Patient With Cytoplasmic MSH2 Expression

**Figure 4:**
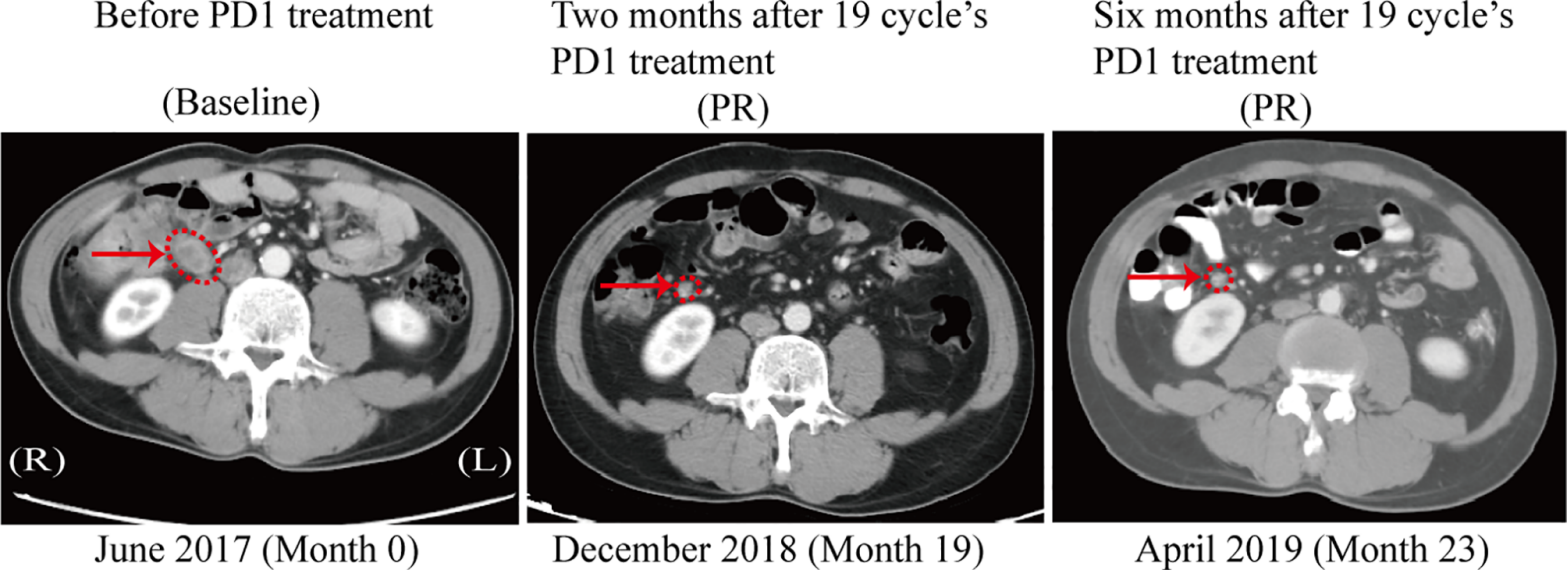
CT scan after PD1 treatment in the patient with MSH2 cytoplasmic staining. A computed tomography (CT) scan revealed the clinical benefits of the PD-1 inhibitor pembrolizumab in the patient with cytoplasmic staining of MSH2. In June 2017, a baseline CT scan showed an enlarged retroperitoneal lymph node (1.8x2.0 cm) with a diagnosis of metastatic colon cancer (arrows). No disease progression was confirmed in this patient 6 months after completion of 19 cycles of immunotherapy, as demonstrated by a stable lymph node size of 0.8x0.8 cm between December 2018 and April 2019 (arrows).

Among patients with tumors showing abnormal MSH2 expression in our cohort, a 45-year-old male (patient 271) was diagnosed with retroperitoneal lymph node metastasis of stage IV colon cancer with rare MSH2 cytoplasmic localization. This patient underwent dissection of distal colon cancer at the age of 33. A cancer family history survey for this patient showed that nine members in three consecutive generations suffered from LS-related cancers (LSRC), including CRC in II6, III2, III5, III7, III11, III13, IV1; pancreatic cancer in II6; and endometrial cancer in III10 (**Figure 2A**). Patient IV1 (case 164) was retrospectively analyzed and identified as having the same MMR pattern as proband III5 (case 271). PCR-MSI confirmed that both patients showing a rare MSH2 cytoplasmic localization were MSI-high, which should be considered an uncommon dMMR pattern. Genetic testing identified this pedigree as LS harboring a novel pathogenic genomic deletion of *EPCAM* and *MSH2* genes. Patient 271 was treated with the PD-1 inhibitor pembrolizumab in combination with capecitabine every 3 weeks for 19 cycles. The patient achieved a clinical partial response (PR) with a 56% reduction in the short axis diameter of the enlarged retroperitoneal lymph node, as revealed by computed tomography (CT) scans after a 19-month course of treatment (**Figure 4**).

## Discussion

In recent years, there has been increasing demand for immunotherapy and LS screening among CRC patients displaying MSI-high or dMMR (27–30). These developments highlight the importance of closely integrating pathologic diagnosis, clinical counseling, and molecular testing for precision medicine approaches to CRC therapy. The purpose of further emphasizing molecular pathology in CRC is to personalize patient treatment and screening of suspected LS patients. In our previous study, we found that a significant proportion of LS patients have LGRs in *MMR* genes in China, a finding that has often been missed by previous next-generation sequencing (22, 31). In the current study, we systematically investigated the diverse mutation patterns of *MSH2* and the clinicopathological characteristics of patients with MSH2 abnormalities in our cohort, especially LGRs, and the corresponding specific genotype-phenotype associations. We found that 12 of 45 (26.7%) LS patients were carriers of *MSH2* and/or *EPCAM* LGRs. Three of 12 (25%) probands with *MSH2/EPCAM* LGRs harbored a rare MSH2 chimeric fusion protein that was detectable in the cytoplasm of tumor cells by IHC. We also provided clinical evidence that a CRC patient harboring cytoplasmic MSH2 fusion proteins was responsive to treatment with immune checkpoint inhibitors.

MMR IHC profiles help discriminate which genes may be deficient in MMR function. Previous studies have indicated that genomic deletion of *MSH2* is a frequent causal event among LS patients (13, 32). One breakthrough study demonstrated that germline deletion of *EPCAM* also leads to inactivation of *MSH2* in families with LS (2). Diverse mutation patterns, especially large genomic deletions/duplications in the *MSH2* and/or *EPCAM* genes, increase the complexity of MSH2 IHC interpretation and molecular testing during LS screening (12, 18, 33–36). dMMR has emerged as a major predictive biomarker for the efficacy of immune checkpoint inhibitors in CRC (19). However, a *post hoc* analysis of clinical trials found that misinterpretation of IHC for MMR proteins was responsible for primary resistance to immune checkpoint inhibitors (21). One rare case of a patient with a *MSH2*/*EPCAM* LGR was reported to show distinct cytoplasmic localization of MSH2 (18). The study also revealed that there was an indication of EPCAM-MSH2 protein fusion rather than artificial nonspecific staining. The cases in this study and others demonstrate that there are still some challenges and pitfalls that pathologists need to avoid. However, because these atypical situations have not been well documented in the literature, they have not been effectively translated into clinical practice guidelines and are easily misinterpreted by pathologists (37–39). In the current study, characterization of three probands with abnormal MSH2 localization by IHC revealed detectable and specific cytoplasmic staining of MSH2 and loss of nuclear MSH2 staining with patchy MSH6 expression in the nucleus. Complementary PCR-MSI tests confirmed an MSI-high pattern suggestive of dMMR in tumors with cytoplasmic MSH2 staining. All three patients were identified as having LS with combined deletion of *MSH2* and *EPCAM*, indicating an interestingly distinct phenotype-genotype association in LS screening. Together, the findings of our study based on a large consecutive CRC cohort demonstrate the pathologic characteristics of this novel MSH2 staining pattern and its possible association with germline mutations in *MMR* genes. Cases in which cytoplasmic MSH2 staining is combined with patchy or weak MSH6 staining in tumor cells are highly suspected to be LS.

IHC staining of MMR proteins should always be interpreted with caution. When interpreting staining as abnormal, pathologists should consider the localization, proportion, intensity and internal control of staining in tumor cells (40). Tumors carrying an *MSH2* germline mutation generally show a complete loss of MSH2 and MSH6 proteins, whereas other uncommon staining patterns, such as MSH2 loss and patchy MSH6 nuclear staining, as well as retained staining of MSH2 and MSH6, have drawn the attention of pathologists and genetic counselors during IHC analysis of MMR proteins (17, 41). The findings of this study highlight another uncommon staining pattern: MSH2 cytoplasmic staining and patchy MSH6 nuclear staining, rather than the absence of staining, in tumor cells. IHC staining of adjacent normal colorectal tissues showed possible MSH2 cytoplasmic expression and nuclear staining. This indicates that a “double hit” in *MSH2* causes inactivation of the normal allele, resulting in its absence in the nuclei of tumor cells. This study expands the pathologic interpretations of MSH2 IHC staining during LS prescreening and immunotherapy testing. The three patients with MSH2 abnormalities were all identified as having LS. The correlation between MSH2 cytoplasmic staining and large genomic deletions in *MSH2/EPCAM* is significant. Therefore, the dMMR pattern of cytoplasmic MSH2 staining and patchy/weak MSH6 nuclear staining should be helpful diagnostically in cases where LS is highly suspected by incorporating LGR analysis of *MSH2/EPCAM*. In addition, on the basis of these LS cases with ambiguous patchy/weak MSH6 staining, we also advocate for the use of four MMR proteins in IHC screens instead of the two-protein (MSH6 and PMS2) staining method (17, 42, 43).

EPCAM is an epithelial cell adhesion molecule located on the cell surface. The protein consists of a signal peptide, extracellular domain (N-terminal), transmembrane domain and cytoplasmic domain (C-terminal). Patients with cytoplasmic staining have a common feature of MSH2 C-terminal fusion with an EPCAM N-terminal fragment of 25 amino acids, including a complete signal peptide (44). The signal peptide is required for cytoplasmic membrane localization of the EPCAM protein. It is possible that the EPCAM signal peptide guides the cytoplasmic membrane translocation of EPCAM-MSH2 fusion proteins in this study. MSH2 and MSH6 in humans form an hMutSα heterodimer and are subsequently imported to the nucleus in a manner dependent on MSH6 nuclear localization sequences (45, 46). The EPCAM-MSH2 fusion proteins may interfere with binding and dimerization with MSH6. This defect in protein interaction in turn results in MSH6 protein instability without heterodimerization with MSH2, as indicated by weak or absent MSH6 staining in the nucleus (17).

In this study, 26.7% of LS CRC patients with MSH2 abnormalities carried pathogenic mutations in *MSH2*/*EPCAM* LGRs, consistent with previous studies (8, 11, 13). *EPCAM* deletions are generally considered to result in promoter hypermethylation and epigenetic silencing of the neighboring *MSH2* gene through transcriptional read-through (2). Our study indicates that nonfunctional chimeric proteins derived from fusion transcripts of *MSH2*/*EPCAM* LGRs were underestimated in LS screening. This was not accidental. Three out of 12 patients with *MSH2*/*EPCAM* LGRs exhibited a similar phenotype of cytoplasmic staining of MSH2. Although the three identified patients from two families harbored two different genomic aberrations, they were found to have the same *EPCAM-MSH2* transcript that involves the fusion of exon 1 of EPCAM and exon 2 of MSH2. A subsequent detailed analysis of breakpoint junctions indicated that the molecular mechanism underlying the novel rearrangements was intrachromosomal recombination mediated by Alu-Alu elements. They harbored different breakpoints in *EPCAM-MSH2* than those reported by Sekine et al. (18). Alu repeats are a family of short interspersed nuclear elements (SINEs) that are prevalent in the human genome. It has been shown that some genes, such as *MSH2* and *EPCAM*, are more prone to LGRs because of the presence of abundant homologous Alu elements (10, 47, 48). Therefore, molecular characterizations of uncommon cases from emerging clinical practice data definitely increase our understanding of LS etiology and contribute to refinements in corresponding genetic diagnostic approaches.

From a clinical standpoint, MMR-deficient CRC cases respond poorly to fluorouracil-based chemotherapeutics and are highly sensitive to immune checkpoint inhibitors (19, 30, 49–52). Misdiagnosis of dMMR and MSI status are the primary factors underlying resistance to immunotherapy among CRC patients (21). A lack of familiarity with the nuances of MMR IHC can lead to interpretive errors (15). Our findings suggest that CRCs with cytoplasmic localization of MSH2 should be considered dMMR and MSI-high. One case also demonstrated that anti-PD-1 immunotherapy shows a durable clinical benefit. Although this is only one example, we believe that, given their dMMR status, such patients should expect good efficacy with immune checkpoint inhibitor therapy. We suggest that PCR-MSI should be endorsed as a complementary test for abnormal patients that show equivocal immunostaining patterns to improve personalized targeted therapy.

In conclusion, our study demonstrates that the rare cytoplasmic MSH2 staining pattern in LS patients should be fully recognized by pathologists and geneticists. Given the specific genotype-phenotype correlation in LS screening, we advocate that all CRC patients with cytoplasmic MSH2 staining in histology should be screened for LGRs of *EPCAM* and *MSH2* in clinical practice.

## Data Availability Statement 

The original contributions presented in the study are included in the article/**Supplementary Material**. Further inquiries can be directed to the corresponding authors.

## Ethics Statement

The studies involving human participants were reviewed and approved by Cancer Hospital of the Chinese Academy of Medical Sciences. Written informed consent to participate in this study was provided by the participants’ legal guardian/next of kin.

## Author Contributions 

JC and JY designed and supervised the overall project. LD, SZ, and YZ compiled and analyzed the data and performed statistical analyses. LD, HL, and LG interpreted the data and drafted the manuscript. JY critically revised the manuscript for intellectual content. All authors contributed to the article and approved the submitted version.

## Funding

This work was supported by grants from the Special Fund of the Chinese Central Government for Basic Scientific Research in Commonweal Research Institutes (2016ZX310024 and 2016ZX310176), Beijing Hope Run Special Fund of the Cancer Foundation of China (LC2017B14), the Non-Profit Central Research Institute Fund of Chinese Academy of Medical Sciences (2019PT310026) and the National Key Research and Development Program (2017YFC1311005). The funders had no role in the study design, data acquisition, analysis, interpretation, writing or submission of the manuscript.

## Conflict of Interest

Author YZ was employed by company Beijing Microread Genetics.

The remaining authors declare that the research was conducted in the absence of any commercial or financial relationships that could be construed as a potential conflict of interest.
