# Supplementary material for: Cytoplasmic MSH2 Related to Genomic Deletions in the MSH2/EPCAM Genes in Colorectal Cancer Patients With Suspected Lynch Syndrome
Source: Front Oncol. 2021 May 14;11:627460. doi: 10.3389/fonc.2021.627460 (PMC8162378; doi:10.3389/fonc.2021.627460)

**Cytoplasmic MSH2 related to genomic deletions in *MSH2/EPCAM* genes in colorectal cancer patients with suspected Lynch syndrome**

Lin Dong, Shuangmei Zou, Xianglan Jin, Haizhen Lu, Ye Zhang, Lei Guo, Jianqiang Cai, Jianming Ying

**Supplementary material contents:**

**Table S1** Primers of cDNA fusion PCR in this study.

**Table S2** Primers of genomic breakpoint PCR in this study.

**Table S3** Frequency of *MSH2* germline mutations in the consecutive colorectal cancer cohort.

**Figure S1** The flowchart of the study.

**Figure S2** The cytoplasmic and nuclear staining of MSH2 protein in proband’s normal colorectal tissue.

**Figure S3** MSI-high in cases with MSH2 cytoplasmic staining in colorectal cancer.

**Figure S4** Confirmation of *EPCAM/MSH2* deletion by MLPA.

**Figure S5** Screening fusion transcript of *EPCAM-MSH2* by cDNA PCR.

**Figure S6** A schematic representation of the long-range PCR strategy to detect breakpoint in case 271.

**Figure S7** A schematic representation of the long-range PCR strategy to detect breakpoint in case 345.

**Table S1** Primers of cDNA fusion PCR in this study

| Primer name | Primer sequence (5’-3’) |
| --- | --- |
| E1M23-1F | GCTCCTCGTGTCCCACTC |
| E1M23-2F | GCACAGAGCGCTAGTCCTTC |
| E1M23-2R | CCACATACCCAACTCCAACC |

**Table S2**  Primers of genomic breakpoint PCR in this study

| Gene | Primer name | Primer sequence (5’-3’) |
| --- | --- | --- |
| *EPCAM* | EF11 | GTTCCAAAACAGCCCCAGCCG |
|  | EF12 | CAGCCTCACTTCGCAGCTTTG |
|  | EF16 | TCTCACTGCAATTCAGGCGATT |
|  | EF20 | TTTCCCAGGCTCAAGCGATG |
|  | EF25 | CCCCAAAGGAAACATCCCTC |
| *MSH2* | MR8 | CCCGCCCTGTTTTAAAGTCTTAT |
|  | MR9 | ACCATATCAAGCACCTAACACAA |
|  | MR12 | CTGCACTTGGTCTGTCCTTTCTT |
|  | MR13 | CATCCCTAAGTGCTGGAACGA |
|  | MR39 | AAGGCAGCTTTCAATCACA |
|  | MR42 | ATTCATACCAGTGAAGCCAAA |
|  | MR44 | GCTGTGATCAACTTTAGGCAAA |
|  | MR45 | AATCCAGGCATGTGATAAAGC |
|  | MR46 | CAAAGGCAGGATAGAACCCAA |
|  | MR47 | TCCAGTTCCTGCCCACGAAG |
|  | MR52 | CTATCCTGTGCCCATAATGACT |
|  | MR54 | CCAGTAAAATTATCACCCCAAC |
|  | MR55 | GGGTCTCTTTCTGTTGCCTA |
|  | MR58 | GCCTCCCAAGTAATTGCAT |
|  | MR59 | TTTCCAACCATGTGCGTGT |
|  | MR60 | AGGCATGTACCTGTAATCCTTG |

**Table S3** Frequency of *MSH2* germline mutations in the consecutive colorectal cancer cohort

|  | LS probands  No. | MSH2-loss  (N=69)  Percent | dMMR  (N=345)  Percent | Total CRCs (N=4195)  Percent |
| --- | --- | --- | --- | --- |
| *MSH2* SNV/indel | 33 | 47.83% | 9.57% | 0.79% |
| *MSH2* LGR | 6 | 8.70% | 1.74% | 0.14% |
| *MSH2/EPCAM* or *EPCAM* LGR | 6 | 8.70% | 1.74% | 0.14% |
| All *MSH2* related mutations | 45 | 65.22% | 13.04% | 1.07% |

CRC, colorectal cancer; dMMR, deficient mismatch repair; Indel, insertion and deletion; LGR, large genomic rearrangement; LS, Lynch syndrome; No., number; SNV, single nucleotide variant.

**Figure S1**  The flowchart of the study


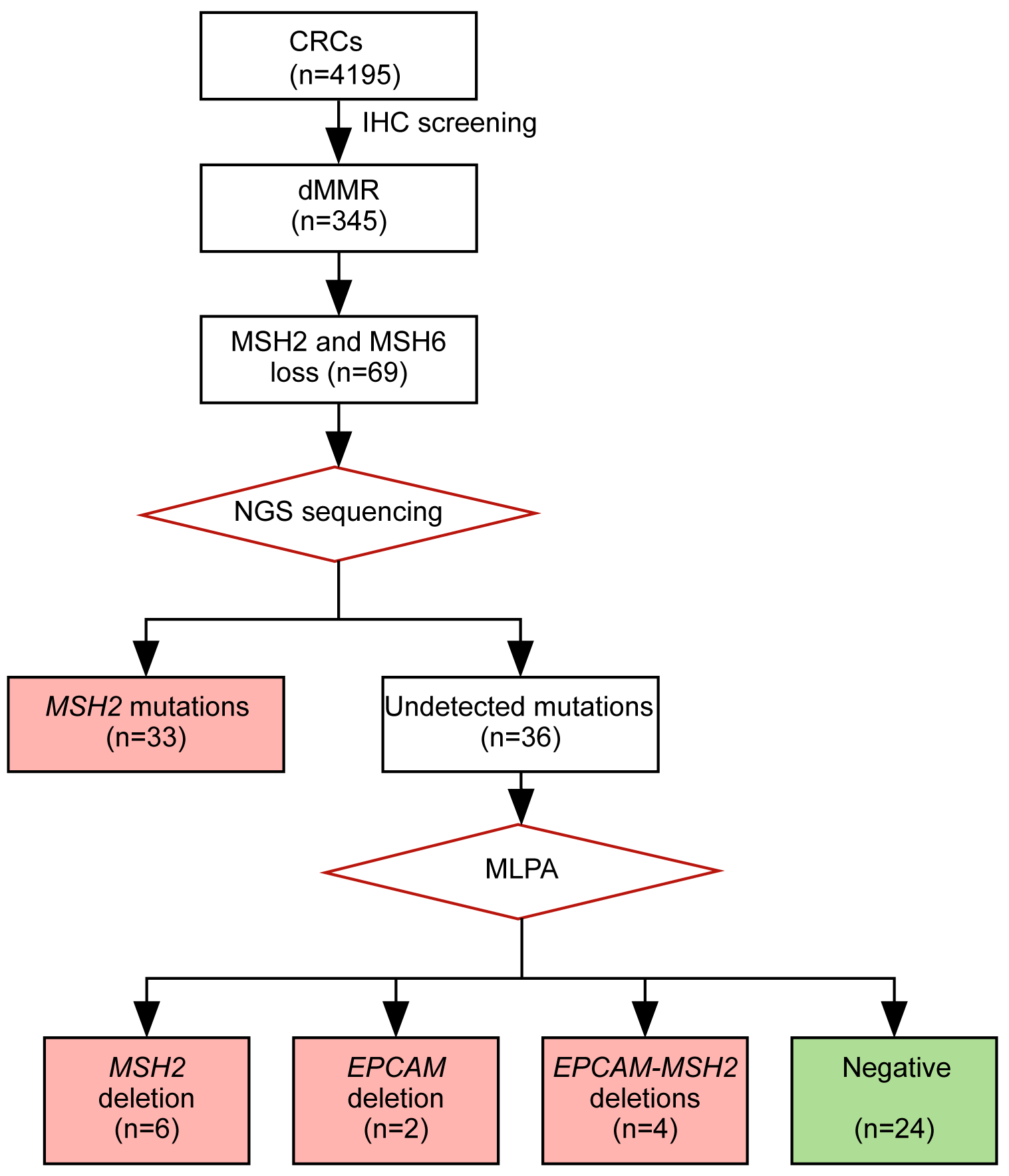


**Figure S2**  The cytoplasmic and nuclear staining of MSH2 protein in normal colorectal tissue. The normal tissue from case 271 with MSH2 cytoplasmic staining in colorectal cancer cells showed MSH2 protein expression suggestive of immnunostaining both in cytoplasmic and nuclear.


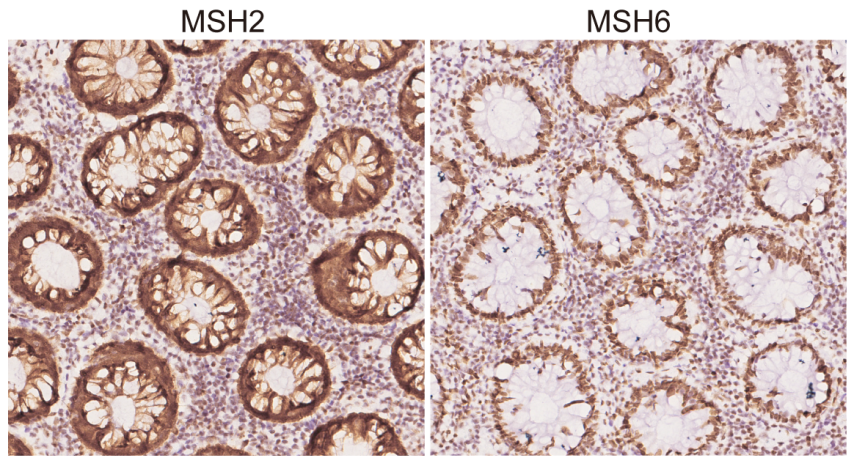


**Figure S3**  MSI-high in cases with MSH2 cytoplasmic staining in colorectal cancer. Amplification profile of fluorescent PCR-based assay in both normal mucosa and tumor tissue from the selected cases showed high microsatellite instability in colorectal carcinomas. The colored areas highlighted the microsatellite shift that displayed instability.


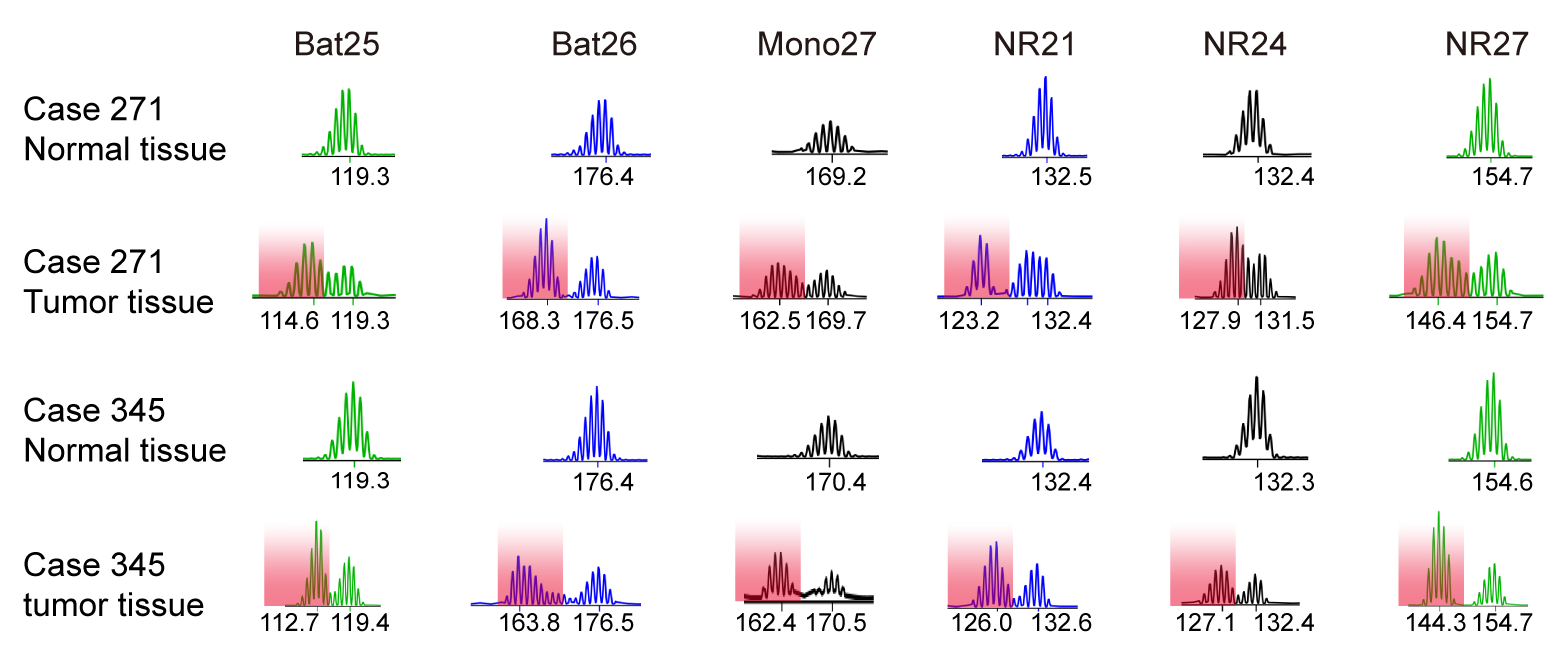


**Figure S4** Confirmation of *EPCAM/MSH2* deletion by MLPA. Multiple ligation-dependent probe amplification (MLPA) testing using P072 kit covering different regions of *EPCAM* and *MSH2* identified the presence of large genomic deletion of *MSH2* and *EPCAM* in case 271 and 345.


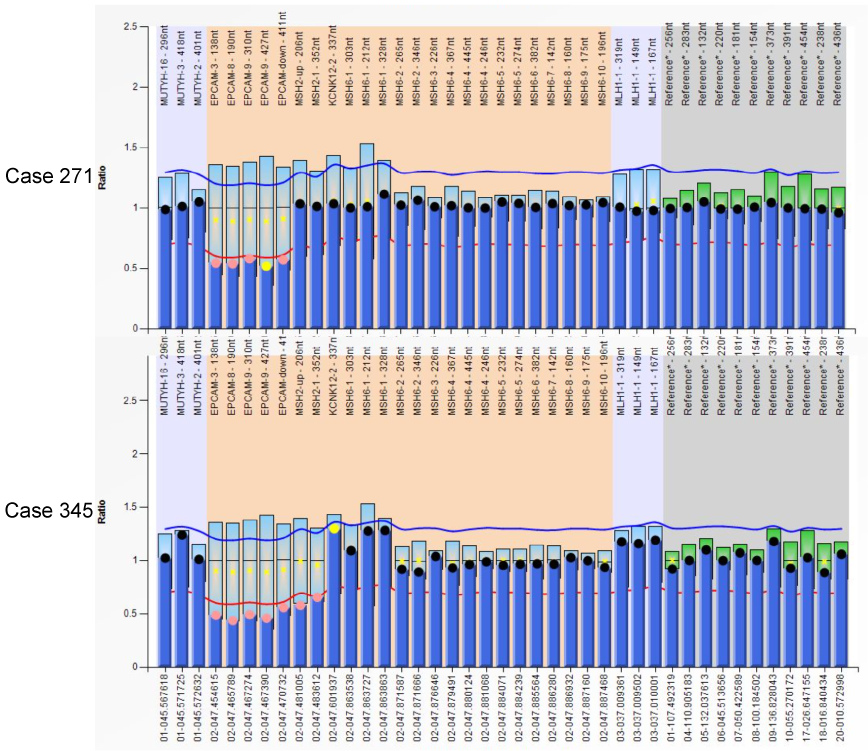


**Figure S5** Screening fusion transcript of *EPCAM-MSH2* by cDNA PCR. (A) PCR products of cDNA amplification from the peripheral blood of the proband were screened by agarose gel electrophoresis. The predicted size band was visualized as lane 1 and 2 for case 271 and 345. (B) The diagram showed the design of primers for detecting fusion transcripts.


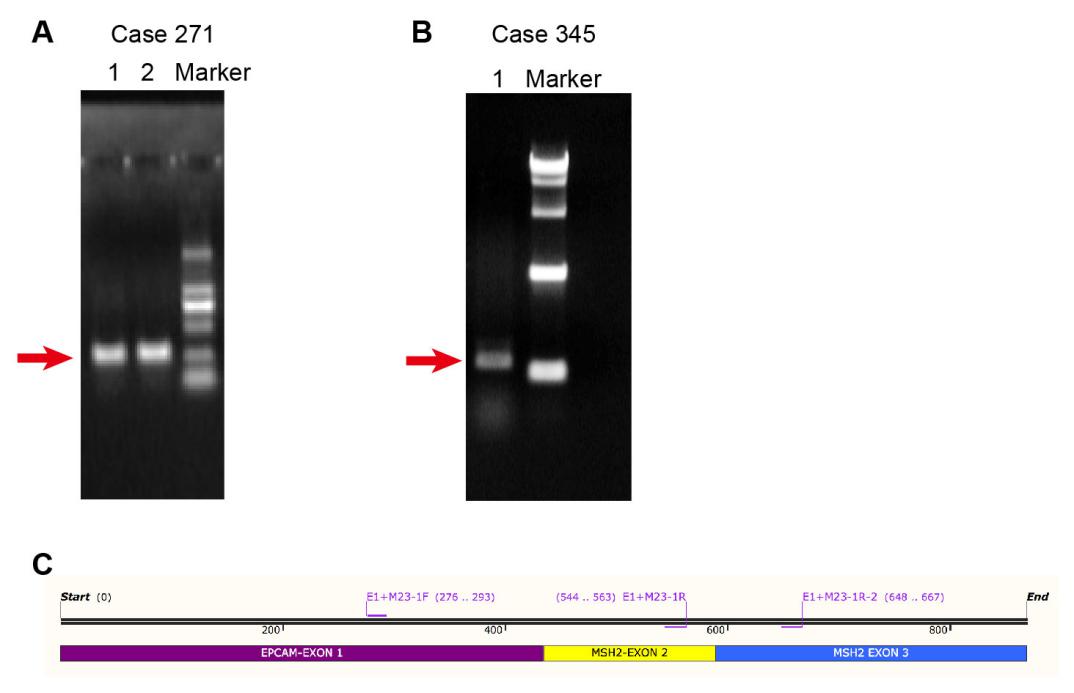


**Figure S6**  A schematic representation of the long-range PCR strategy to detect breakpoint in case 271. (A) Series of primers for screening the breakpoint of *EPCAM* and *MSH2* were designed covering different regions of *EPCAM* intron1 and *MSH2* upstream. (B) Different PCR products from the designated primer pairs were visualized in agarose gel electrophoresis. The expected size band amplified by primer pairs of EF25 and MR54 was selected for sanger sequencing.


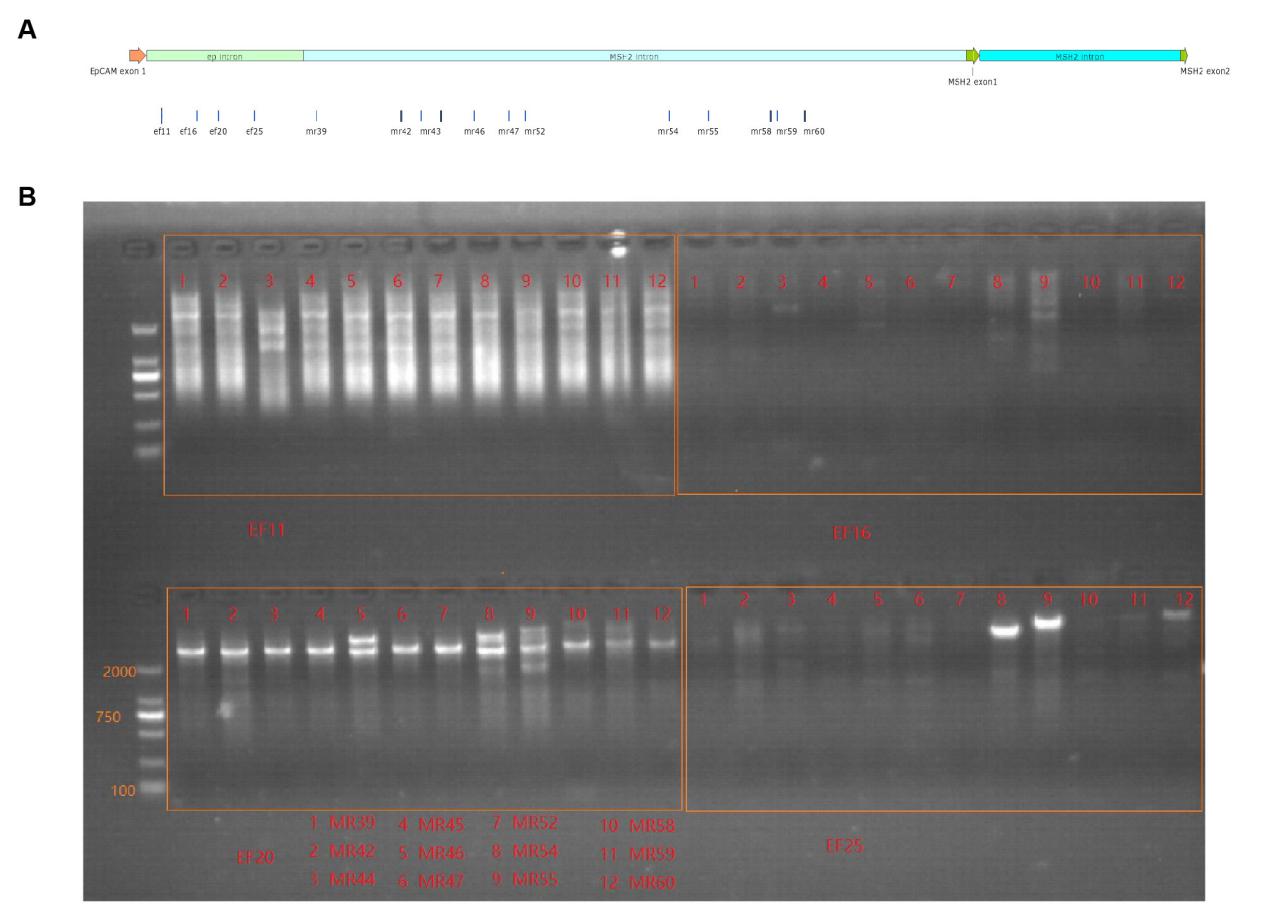


**Figure S7**  A schematic representation of the long-range PCR strategy to detect breakpoint in case 345. (A) Series of primers for screening the breakpoint of *EPCAM* and *MSH2* were designed covering different regions of *EPCAM* intron1and *MSH2* intron1. (B) Different PCR products from the designated primer pairs were visualized in agarose gel electrophoresis. The expected size band amplified by primer pairs of EF11 and MR8 as shown in the lane 1 of case 345 was selected for sanger sequencing.


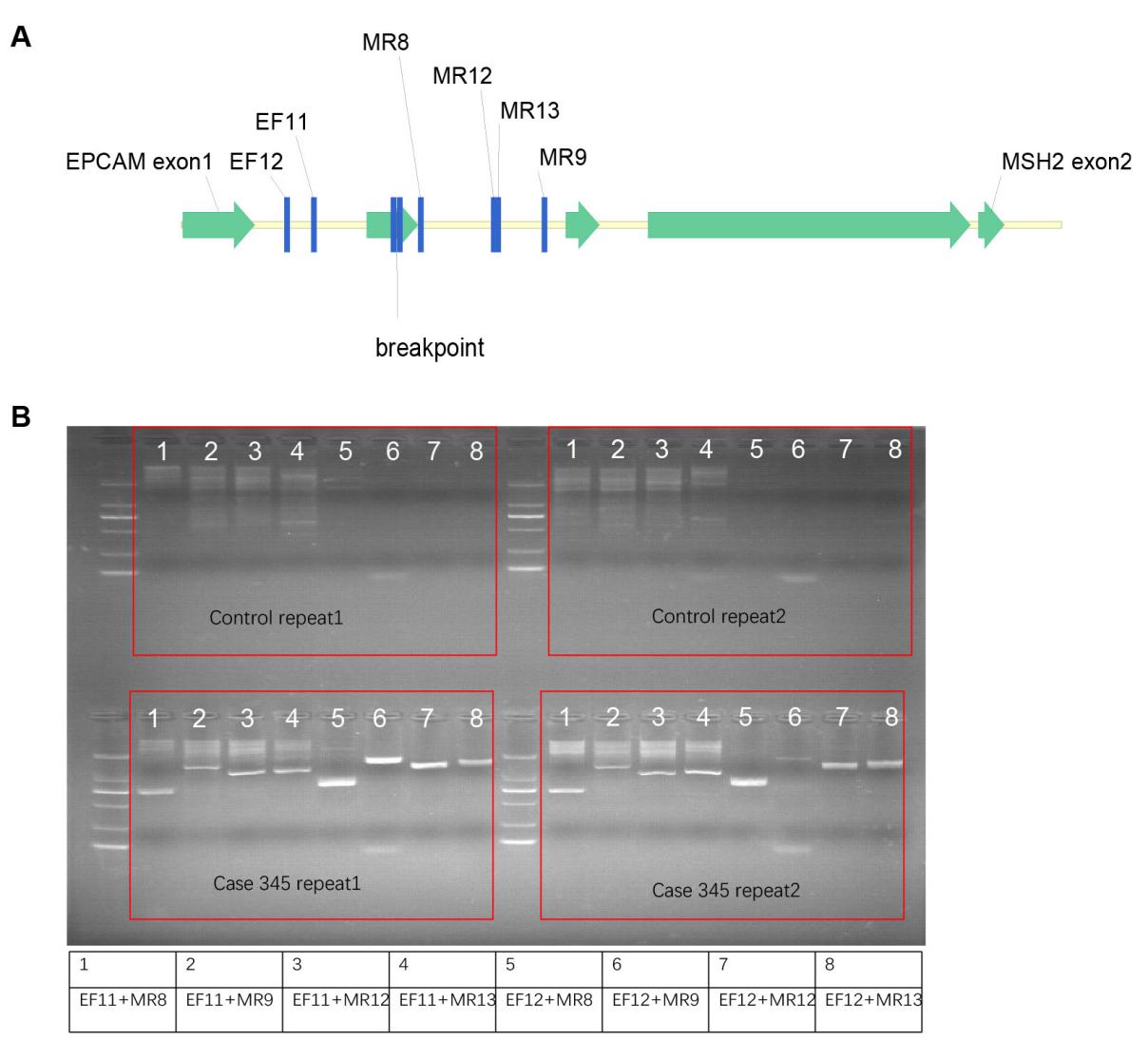

Supplement: Supplementary file 1 [file DataSheet_1.docx]
